# The effect of using NHS number as the unique identifier for patients who self-harm: a multi-centre descriptive study

**DOI:** 10.1186/1745-0179-3-16

**Published:** 2007-09-21

**Authors:** Jayne Cooper, Elizabeth Murphy, Helen Bergen, Deborah Casey, Keith Hawton, David Owens, Rachael Lilley, Rachael Noble, Navneet Kapur

**Affiliations:** 1 Centre for Suicide Prevention, Division of Psychiatry, 7th Floor Williamson Building, University of Manchester, Oxford Road, Manchester M13 9PL, UK; 2 Centre for Suicide Research, University of Oxford, Department of Psychiatry, Warneford Hospital, Oxford OX3 7JX, UK; 3 Academic Unit of Psychiatry and Behavioral Sciences, 15 Hyde Terrace, University of Leeds, Leeds LS2 9LT, UK

## Abstract

**Background:**

Processing personal data for research purposes and the requirement of anonymity has been the subject of recent debate. We aimed to determine the proportion of individuals who present to emergency departments with non-fatal suicidal behavior where an NHS number has been successfully traced and to investigate the characteristics of patients associated with non-capture.

**Method:**

This was a descriptive study of people attending after self-harm using allocation of NHS numbers as main outcome measurement. Data from the Multicentre Monitoring of Self-Harm Project from 3 centres in England were used to identify consecutive patients (N = 3000) who were treated in six emergency departments in Oxford, Manchester and Leeds in 2004 and 2005 following self-harm.

**Results:**

NHS number was available between 55–73% of individuals across centres. Characteristics associated with non-recording of NHS number in more than one centre included those from ethnic minority groups (Oxford: chi-squared statistic = 13.6, df = 3, p = 0.004; Manchester: chi-squared statistic = 13.6, df = 3, p ≤0.001) and the homeless or living in a hostel or other institution (Oxford: chi-squared statistic = 40.9, df = 7, p = <0.001; Manchester: chi-squared statistic = 23.5, df = 7, p = 0.001). Individual centre characteristics included being of male gender (Leeds: chi-squared statistic = 4.1, df = 1, p = 0.4), those under 25 years (Oxford: chi-squared statistic = 10.6, df = 2, p = 0.005), not being admitted to general hospital (Leeds: chi-squared statistic = 223.6, df = 1, p ≤0.001) and using self-injury as a method of harm (Leeds: chi-squared statistic = 41.5, df = 2, p ≤0.001).

**Conclusion:**

Basing research studies on NHS number as the unique identifier, as suggested by the Data Protection Act 1998 and the Patient Information Advisory Group, would exclude some of the most vulnerable groups for further self-harm or suicide. This bias may also affect other research registers.

## Background

The Data Protection Act 1998 [1] brings into the UK the European Directive on the processing of personal data [2]. The Act raises requirements for patient consent and anonymization of data for research purposes. However explicit consent is not always practical or possible; patient identification is often necessary in medical research to ensure the integrity of the data and accurate record linkage [3]. A strategy which may overcome some of these difficulties is pseudonymization of data, which involves holding personal identifying data separate from substantive data, but preserving a key which allows remerging. The Patient Information Advisory Group [4] assesses applications for Section 60 approval under the Health and Social Care Act 2001 [5] for individual research projects requiring patient identification but where individual consent cannot be achieved. They suggested one way of achieving pseudonymization of data is the use of the NHS number as the sole identifier. In order for this system to be successful, identification of patients' NHS numbers needs to be accurate and comprehensive with an avoidance of the exclusion of selective groups [6]. Currently, NHS trusts are required to trace NHS numbers for 95% of their 'active' patients (i.e. patients admitted or with active follow up care). However, not all patients who attend the emergency departments will become 'active' patients which may compromise the comprehensiveness of the tracing process.

Self-harm is a major public health problem [7-9] and monitoring is recommended in the National Suicide Prevention Strategy for England [10]. Individual identifiers are required if information on rates of self-harm, repetition and subsequent suicide are to be calculated. Risk analysis of individual projects about restrictions on the use of personal data has been recommended [11]. We have assessed the bias that might be introduced by a system based solely on NHS numbers. We sought to determine the uptake from three well established self-harm registers to identify those people likely to be excluded.

## Method

One thousand consecutive individuals who presented following self-harm to emergency departments in each of three centres in Oxford and Manchester from 1^st ^January 2004 and in Leeds from 1^st ^October 2004 were included in the study. We included patients who did not wait for treatment. These centres contributed to the multicentre monitoring of self-harm project described in detail elsewhere [12]. Self-harm was defined as intentional self-poisoning or self-injury, irrespective of motivation [13]. The method of NHS number allocation varied between centres:

### Oxford

NHS numbers were determined via the emergency department computer system. This system had general connectivity to the main Patient Master Index. Therefore NHS capture was not necessarily limited to 'active' patients.

### Manchester

For the study period NHS number allocation was determined via a single batch trace to the National Strategic Tracing Service of all self-harm patients.

### Leeds

The computer systems at the Leeds hospitals during the study period, now updated, were stand-alone systems. Therefore NHS numbers were only available for patients who were entered on to the main Patient Administration System. Patients were entered thus if they were termed 'active' i.e. admitted, or had follow-up treatment arranged such as outpatient appointments or clinic follow-up. Once patient details were on the system their NHS numbers were traced online via the National Strategic Tracing Service.

## Results

An NHS number was identified in 73.1%, 72.8% and 55.1% for the individual centres in Oxford, Manchester and Leeds respectively. Table 1 shows data from all centres and Table 2 from Oxford and Manchester only and the association between socio-demographic and clinical variables with non capture of NHS number.

**Table 1:** Characteristics of self-harm patients associated with non-capture of NHS number: all three centres

		Oxford (N = 1000)			Manchester (N = 1000)			Leeds (N = 1000)		
		N	%*	Statistic	N	%*	Statistic	N	%*	Statistic
Total attendances without NHS number		269	27		272	27		449	45	
Gender	Female	159	26	X^2 ^= 1.6	143	25	X^2 ^= 2.0	241	42	X^2 ^= 4.1
	Male	110	29	df = 1	129	30	df = 1	208	49	df = 1
				P = 0.21			P = 0.16			P = 0.04
Age	Up to 24	109	32	X^2 ^= 10.6	92	28	X^2 ^= 2.3	144	46	X^2 ^= 4.7
	25 – 54	148	26	df = 2	168	28	df = 2	284	46	df = 2
	55 Plus	12	15	P = 0.005	11	19	P = 0.32	20	32	P = 0.10
Method of self-harm	Self-poisoning	206	27	X^2 ^= 0.8	219	27	X^2 ^= 0.0	307	40	X^2 ^= 41.5
	Self-injury	48	30	df = 2	45	27	df = 2	113	67	df = 2
	Both	15	25	P = 0.67	7	27	P = 1.0	29	45	P < 0.001
Time of presentation	9 am – 5 pm	80	27	X^2 ^= 0.01	92	32	X^2 ^= 4.1	129	48	X^2 ^= 1.5
	Out of hours	167	27	df = 1	177	25	df = 1	318	44	df = 1
				P = 0.91			P = 0.04			P = 0.22
Admitted to general	Yes	226	26	X^2 ^= 1.8	125	27	X^2 ^= 0.4	202	29	X^2 ^= 223.6
hospital	No	43	32	df = 1	96	25	df = 1	247	80	df = 1
				P = 0.18			P = 0.55			P < 0.001
No fixed abode	NFA	11	32	X^2 ^= 0.6	19	86	X^2 ^= 40.2	12	60	X^2 ^= 1.9
	Address	252	26	df = 1	250	26	df = 1	429	44	df = 1
				P = 0.43			P < 0.001			P = 0.17

Completeness of independent variables: data at all centres at least 97% complete for "gender", "age" and "method of harm"; "time of presentation" at least 85%; "admitted to general hospital" at least 92%; "no fixed abode" at least 86%.

* percentage of category by variable with no NHS number

**Table 2:** Characteristics of self-harm patients associated with non-capture of NHS number: Oxford and Manchester

		Oxford (N = 1000)			Manchester (N = 1000)		
		N	%*	Statistic	N	%*	Statistic
Total attendances without NHS number		269	27		272	27	
Previous self-reported self-harm	Yes	103	21	X^2 ^= 16.6	126	25	X^2 ^= 0.1
	No	96	35	df = 1	68	26	df = 1
				P < 0.001			P = 0.75
Alcohol use at time of self-harm	Yes	113	23	X^2 ^= 4.2	98	24	X^2 ^= 0.8
	No	116	29	df = 1	86	27	df = 1
				P = 0.04			P = 0.37
Living arrangement	NFA	11	32		9	75	
	Hostel or institution	25	45		20	36	
	Alone	29	19	X^2 ^= 40.9	40	24	X^2 ^= 23.5
	Spouse/partner	76	29	df = 7	48	23	df = 7
	Parent/sibling	54	24	P < 0.001	36	23	P = 0.001
	Friends/other relatives	18	41		17	26	
	Children only	7	10		6	14	
	Student halls	18	53		8	31	
Ethnicity	White	165	25	X^2 ^= 13.6	164	23	X^2 ^= 25.6
	Black	7	50	df = 3	6	27	df = 3
	South Asian	8	62	P = 0.004	20	56	P < 0.001
	Other	7	37		11	48	

Completeness of independent variables: "previous self-reported self-harm" Oxford 76%; M/cr 77%, "alcohol use at time of self-harm" Oxford 89%; M/cr 74%, living arrangement Oxford 88%; M/cr 73% and "ethnicity" Oxford 70%; M/cr 79%.

* percentage of category by variable with no NHS number

In Oxford non capture of NHS number was more likely in the younger age groups, those living in student halls and hostels or other institutions and those of non-white ethnic group, particularly of South Asian origin. Those with a previous history of self-harm and who used alcohol at the time of harm were more likely to have a known NHS number.

In Manchester non capture of NHS number was associated with presentation during normal working hours, those of no fixed abode, living in hostels or other institutions and ethnic minority groups, particularly those of South Asian origin.

In Leeds non capture of NHS number was associated with males and self-injury as a method of harm. Those of no fixed abode were proportionately less likely to have an NHS number, although this association did not reach significance. Those who were admitted to general hospital were more likely to have a known NHS number.

## Discussion

In our sample, NHS number capture was unsuccessful in one third of self-harm attendances overall. Those of minority ethnic groups, particularly of South Asian origin, the homeless and those living in hostels or other institutions, would be under represented on the self-harm database if the sole identifier was the NHS number. Amongst these excluded groups are those at high risk of further self-harm and suicide. For example, young South Asian women have the highest rates of self-harm compared to young white women [14-16], and homelessness has been found to be associated with increased mortality and suicidal behavior [17,18]. In individual centres, male gender, younger age and self-injury as a method of harm were less likely to have an NHS number. These factors are also important predictors of increased risk [19-21].

Differences between centres may in part be explained by the extent of connectivity of their computer systems. Rate of capture based on NHS number are generally lower in emergency departments than for other departments within acute trusts. In Leeds, where the emergency departments had stand alone computer systems, non-admitted patients to general hospitals were the least likely group to have an NHS number allocated (only 20%), presumably because they would be less likely to be classified by the trust as 'active'. Funding priorities for Trusts mean there is less incentive for them to trace the NHS number for 'inactive' patients. If the computer system in the emergency department is connected to the central hospital computer records system then it is possible to trace patients who are not necessarily 'active'.

Connecting for Health is an ambitious government IT programme in the UK, specifically aimed at supporting a unified NHS, intended to be introduced nationally over the next five years [22]. Developments in NHS IT infrastructure [23] and increased NHS number allocation may improve NHS number uptake. Even so, within the central hospitals computer records system there are groups that by default will not have an NHS number. The Connecting for Health website does not, for example, make a specific reference to adding new NHS numbers for people who are homeless or of no fixed abode. There are also no current plans to date to allocate NHS numbers to temporary overseas visitors, legal or illegal.

The current arrangements for the recording of NHS number exclude vulnerable people who attend emergency departments following self-harm. At present we cannot recommend, on the basis of the findings of this study, the pseudonymization of research data using NHS numbers as the sole identifier of those who self-harm. The shortfall in recording practice may be a problem for other medical registers; it is certainly a problem for self-harm registers and this has important implications for the monitoring of suicidal behavior and suicide prevention.

## Competing interests

The author(s) declare that they have no competing interests.

## Authors' contributions

All authors had input into the design of the study. KH leads the multicentre project. JC, EM, HB, DC, RL & RN oversaw the collection of the data, JC and EM planned the analysis with advice from NK, and was carried out by EM and JC. JC and NK interpreted the results and HB, DC, RL, RN and DO commented on the analysis, JC wrote original draft. All authors have read and approved the final manuscript. JC is guarantor of the paper
